# EDGEdb: a transcription factor-DNA Interaction database for the analysis of *C. elegans *differential gene expression

**DOI:** 10.1186/1471-2164-8-21

**Published:** 2007-01-18

**Authors:** M Inmaculada Barrasa, Philippe Vaglio, Fabien Cavasino, Laurent Jacotot, Albertha JM Walhout

**Affiliations:** 1 Program in Gene Function and Expression and Program in Molecular Medicine, University of Massachusetts Medical School, Worcester, MA 01605, USA; 2 Modul-Bio, 232 Boulevard Sainte-Marguerite, 13009 Marseille, France

## Abstract

**Background:**

Transcription regulatory networks are composed of protein-DNA interactions between transcription factors and their target genes. A long-term goal in genome biology is to map protein-DNA interaction networks of all regulatory regions in a genome of interest. Both transcription factor -and gene-centered methods can be used to systematically identify such interactions. We use high-throughput yeast one-hybrid assays as a gene-centered method to identify protein-DNA interactions between regulatory sequences (*e.g*. gene promoters) and transcription factors in the nematode *Caenorhabditis elegans*. We have already mapped several hundred protein-DNA interactions and analyzed the transcriptional consequences of some by examining differential gene expression of targets in the presence or absence of an upstream regulator. The rapidly increasing amount of protein-DNA interaction data at a genome scale requires a database that facilitates efficient data storage, retrieval and integration.

**Description:**

Here, we report the implementation of a *C. **e**legans ***d**ifferential **g**ene **e**xpression **d**ata**b**ase (EDGEdb). This database enables the storage and retrieval of protein-DNA interactions and other data that relate to differential gene expression. Specifically, EDGEdb contains: i) sequence information of regulatory elements, including gene promoters, ii) sequence information of all 934 predicted transcription factors, their DNA binding domains, and, where available, their dimerization partners and consensus DNA binding sites, iii) protein-DNA interactions between regulatory elements and transcription factors, and iv) expression patterns conferred by regulatory elements, and how such patterns are affected by interacting transcription factors.

**Conclusion:**

EDGEdb provides a protein-DNA -and protein-protein interaction resource for *C. elegans *transcription factors and a framework for similar databases for other organisms. The database is available at .

## Background

Differential gene expression is governed, at least in part, by protein-DNA interactions between transcription factors (TFs) and their target genes. Together, such protein-DNA interactions can be modeled into transcription regulatory networks that describe the logic underlying the development, function, and pathology of a system of interest [1,2]. Two complementary strategies are currently being used to identify protein-DNA interactions: TF-centered approaches, where the DNA sequences that interact with a TF or set of TFs of interest are identified; and gene-centered methods that identify the TFs that interact with a regulatory DNA sequence or set of DNA sequences of interest (*e.g*. gene promoters)[2]. Previously, we developed a high-throughput yeast one-hybrid system for the gene-centered mapping of protein-DNA interactions between gene promoters or small *cis*-regulatory elements and TFs in the nematode *Caenorhabditis elegans *[3,4]. So far, we have identified 605 protein-DNA interactions between 115 gene promoters and 176 TFs [3,5-7]. In addition, we identified protein-DNA interactions between several small *cis*-regulatory DNA elements and TFs (*i.e*. to identify consensus TF binding sites). Several TFs bind DNA as dimers and such TF-TF dimers are being systematically identified by high-throughput protein-protein interaction mapping efforts [8-10]. Longer term, TF dimer information needs to be incorporated in transcription regulatory network models. We ultimately aim to generate a protein-DNA interaction map between all regulatory DNA elements and TFs in the *C. elegans *genome [2].

Most *C. elegans *research data are collected and maintained in the database WormBase [11,12]. This database provides an indispensable resource for *C. elegans *researchers and contains information about, for instance, the genome sequence and annotation, bioinformatic protein domain annotation, mutants, phenotypes, *etcetera*. However, this database is not as convenient for the retrieval and manipulation of comprehensive protein-protein and protein-DNA interaction datasets, as well as for the storage of manually curated annotations of families of genes (*e.g*. TFs). Several databases have been developed for the storage of data related to gene regulation, including Transfac [13,14] and Jaspar [15,16] that contain information about TF binding sites. Oreganno is a database that contains collections of regulatory sequences and TF binding sites for a variety of organisms [17,18]. The data in this database are not extensively curated as researchers are free to enter and manipulate data themselves. Hence, this database contains many types of experimental data, which does have clear advantages. However, it is not convenient to navigate and download protein-DNA interactions involving TFs and precisely defined genomic sequences.

The rapidly increasing amount of gene-centered protein-DNA interaction data and how such interactions affect differential gene expression requires a frequently updated and curated database for optimal data storage, retrieval and integration. Here, we report the implementation of a *C. **e**legans ***d**ifferential **g**ene **e**xpression **d**ata**b**ase, or EDGEdb, a database that is specifically tailored for the storage, retrieval and integration of physical interactions between *C. elegans *TFs and precisely defined genomic regulatory DNA sequences.

## Construction and content

In gene-centered yeast one-hybrid assays, two types of "DNA baits" are used to identify interacting TFs: single copy *C. elegans *genomic sequences such as gene promoters, and artificial baits such as (putative) *cis*-regulatory DNA elements [5]. EDGEdb contains information about i) DNA bait sequences and genomic coordinates; ii) all 934 predicted *C. elegans *TFs [19], *i.e*. their DNA binding domain, and, where available, dimerization partners and consensus binding sites; iii) protein-DNA interactions between DNA baits and TFs; and iv) where available, the transcriptional consequences of such protein-DNA interactions (see below). In total, the database contains 605 protein-DNA interactions between 115 *C. elegans *gene promoters and 176 TFs. In addition, the database contains protein-DNA interactions for 3 short DNA sequences that were either found by us or by other groups (referred to as "artificial baits", see *e.g*. ZTF-2 or DAF-12). Finally, the database contains 24 TF protein-protein dimer interactions. The regulation of several *C. elegans *genes by specific TFs has been documented in the literature, and we have included some of these in EDGEdb. However, we have not included cases where regulation is reported but where the genomic sequences involved have not been precisely mapped (*i.e*. sequences other than the promoter could be involved, the promoter sequence is not available, or the regulatory interaction is indirect). In the future, we aim to incorporate additional interaction data obtained by other laboratories as they become available. We encourage researchers to send us their data on the EDGEdb homepage. In addition, we will continue to incorporate TF dimerization data, both obtained by our own yeast two-hybrid assays and obtained by other groups.

EDGEdb is implemented on a Jboss application server using an Oracle Database. We used the Bio::DB::GFF [20] database schema to handle genomic sequence information. This schema is loaded with a filtered version of the GFF annotations and DNA sequences in FASTA format from WormBase [21]. Filtered GFF annotations only include gene structure information (5'UTR, exons, introns, 3'UTR), and operon and gene locations. We have also included a WormBase geneID file [22] that provides relationships between WormBase gene name, locus/CGC name and the sequence name. Since only the WormBase ID is always maintained (*i.e*. the names and sequence may change as gene models and functional annotations improve), we use it as a key for the annotations. In addition to the Bio::DB::GFF schema, the database is composed of 26 tables that contain information about the protein-DNA interaction experiments, DNA baits, TFs, TF dimers and consensus binding sites, expression data, and publications. The interfaces have been developed using J2EE/JSP technology, except for the genome display that uses a modified version of GBrowse [23,24]. The genome sequence can be updated using new GFF annotations from WormBase. A series of Perl scripts can then "remap" the DNA baits to their new genomic position. EDGEdb will be updated with every WormBase freeze release (that is every 10 versions). For optimal utility, EDGEdb is linked to both WormBase [11,12] and Worfdb, the database that contains information regarding the collection of *C. elegans *cloned open reading frames (ORFs) in the ORFeome project [25,26].

## Utility and discussion

### EDGEdb description

**Figure 1:**
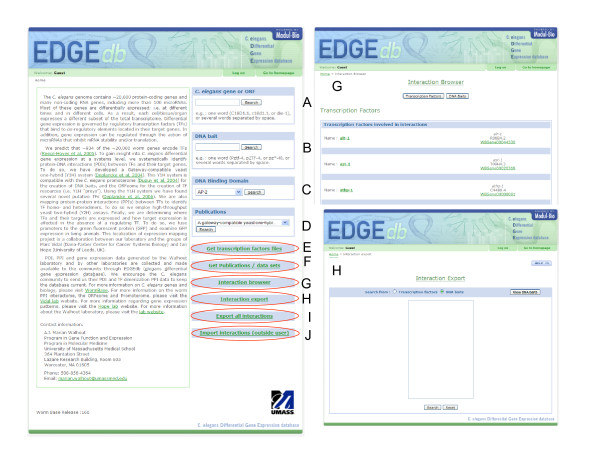
**EDGEdb home page**. This page contains an introduction to the database (left). The home page also provides links to several tools to explore and download the data (right): (A) Text field to search for *C. elegans *genes. (B) Text field to search for DNA baits (*e.g*. a promoter). (C) Scroll down menu to retrieve TFs by DNA binding domain. (D) Scroll down menu to retrieve publications and associated experiments. (E) Link to the collection of *C. elegans *TFs. (F) Yeast one -and two-hybrid datasets and pdf files of corresponding publications. (G) Interaction browser. (H, I) Export tools. (J) Import interaction tool for outside users. The WormBase release used is specified at the bottom left corner of the page.

The EDGEdb home page allows four types of queries: i) individual genes or lists of "space separated" genes can be searched (*e.g. daf-3*, Figure 1A); ii) a second text field is available for DNA bait names (*e.g. Pdaf-3*, the promoter of the *daf-3 *gene, Figure 1B); iii) a scroll down menu in the DNA binding domain text field allows the retrieval of TFs by DNA binding domain (*e.g*. AP-2, Figure 1C), and iv) a search by publication links to experiments and associated excel files of individual studies (Figure 1D, F). An "interaction browser" lists DNA baits and TFs for which interactions are available and facilitates the retrieval of stored interactions (Figure 1G). The "interaction export" interface allows the query and download of interactions from different publications for a set of either DNA baits or TFs (Figure 1H). The "export all interactions" tool allows the download of all available interactions in the database (Figure 1I). These features are designed to facilitate the retrieval and integration of interactions from the different available datasets included in EDGEdb. They allow the user to quickly identify which DNA baits have been assayed so far, to identify the TFs retrieved, and to specifically obtain and download interactions for all or a set of DNA baits or TFs of interest from any of the datasets. Access to the complete collection of predicted *C. elegans *(worm) TFs (wTF2.0)[19] is available through the home page (Figure 1E). This resource will be updated as new TFs are identified and as gene predictions are updated [7]. To facilitate outsider data submission, we have implemented a page for data upload (Figure 1J). After manual curation, the data will be incorporated into EDGEdb.

### EDGEdb gene page

**Figure 2:**
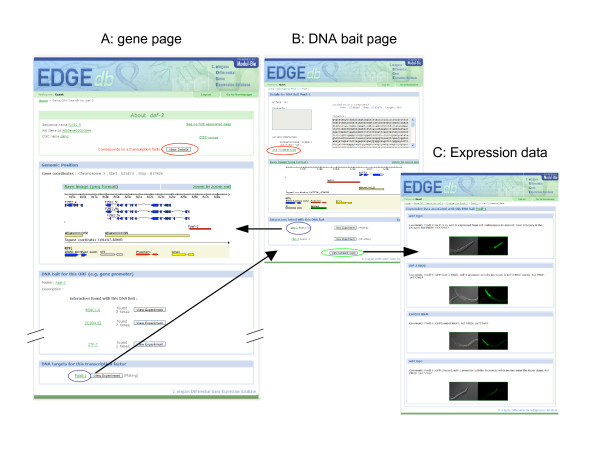
**EDGEdb gene, DNA bait and expression pages**. (A) EDGEdb *daf-3 *gene page. The top section contains the different *daf-3 *gene identifiers, each of which is linked to WormBase. In addition, it contains a link to Worfdb, and the detailed TF page (red circle). The middle section contains the genome view of the *daf-3 *gene. The third section lists the DNA baits associated with *daf-3 *(*i.e*. the *daf-3 *promoter, *Pdaf-3*) and their corresponding yeast one-hybrid interactors (*i.e*. TFs that bind to *Pdaf-3*). The bottom section includes the DNA baits bound by the TF DAF-3 (blue circle). By clicking this one goes to the *Pmdl-1 *DNA bait page. (B) EDGEdb DNA bait page for *Pmdl-1*. This page displays the DNA bait sequence and genomic coordinates; IDs of the gene corresponding to the DNA bait and link to the gene page (red circle). The middle section contains the genome browser. In the bottom section, the list of the interactors found with the DNA bait and links to the relevant experiments and gene pages are shown (blue circle). By clicking this link one goes back to the *daf-3 *gene page. A link to the page containing the expression patterns driven by the DNA bait is included (green circle). By clicking this one goes to the expression page for *Pmdl-1*. (C) Expression data page for *Pmdl-1*. From top to bottom: GFP expression driven by *Pmdl-1 *in wild type animals, in *daf-3(RNAi) *animals, in control RNAi animals, and in dauer animals. See Deplancke et al. [5] for details.

A gene page (Figure 2A) can be accessed by searching for a sequence name (*e.g*. F25E2.5), CGC (*Caenorhabditis *Genetics Center) name (*e.g. daf-3*) or WormBase identifier (*e.g*. WBGene00000899). The page displays alternative names used for the gene (Figure 2A, top), the genomic coordinates, and a genome view of gene models and available DNA baits (Figure 2A, middle). All elements in the genome view link to their respective gene or DNA bait page (see below). The next section (Figure 2A, third section) displays a list of DNA baits associated with the gene (*e.g*. gene promoter sequences). DNA bait names link to the respective DNA bait pages (see below). Additional features are available for genes that encode TFs, including a link to the TF page (see below) (Figure 2A top, red circle), and a list of DNA targets the TF interacts with (Figure 2A bottom, blue circle). Finally, the detailed experiment for each interaction is retrieved through the "view experiment" button (Figures 2A bottom).

### EDGEdb transcription factor page

There is a page for each of the 934 predicted TFs describing its DNA binding domain, dimerization partners, and consensus binding site, where available [19](data not shown). In addition, links to Jaspar [15,16], Transfac [13,14] and WormBook [27], that may contain additional TF binding site information, are provided. The experiment pages for the protein-DNA interactions are accessed through the "view experiment" button. References to the publications where protein-protein interactions and consensus binding sites were reported are included. The DNA baits bound by the TF are listed at the bottom of the page, and links to the corresponding experiments and export tools (see below) are included.

### EDGEdb DNA bait Page

In EDGEdb, genomic sequences that correspond to DNA baits are specified with their chromosome coordinates, and the corresponding sequence is displayed on the page (Figure 2B). The DNA bait name relates to the downstream gene (*e.g. Pmdl-1*, the promoter of the gene *mdl-1*, Figure 2B). The DNA bait position with respect to the gene model may change as gene models are updated. TFs that interact with a DNA bait are listed at the bottom of the page (Figure 2B, bottom) and links to the respective gene page (Figure 2B, blue circle) and relevant experiments are provided. Finally, where available, expression patterns conferred by DNA baits and changes in these patterns in the absence of an interacting TF can also be accessed through this page (Figure 2B green circle, Figure 2C). Several artificial DNA baits are also included in the database (*e.g*. P2_multimer).

### EDGEdb experiment page

Each DNA bait used is linked to at least one experiment page that details the information about the type of experiment that was carried out and the list of interactors found. For interactions reported in the literature, "literature" is specified as the source of the interaction, and the PubMed ID is provided (*e.g*. the MDL-1/MLX-1 dimerizing protein-protein interaction reported on the corresponding TF pages is referred to as PMID: 9764821).

### Export of protein-DNA interaction data

A user can download excel files containing protein-DNA interaction information from the TF, DNA bait and experiment pages. The user can also download text files containing the sequence of DNA baits bound by a TF from the TF pages. Additionally, the home page links to an "interaction export" page and to an "export all interactions" tool that allow query and download of all or a subset of interactions.

### EDGEdb significance

Several features make EDGEdb a valuable resource for both the *C. elegans *and systems biology of gene expression/transcription communities. First, as more interaction data become available, extracting information for the user's favorite set of genes or TFs will become time consuming. EDGEdb allows the efficient retrieval and integration of different protein-DNA interaction and TF-TF dimerization datasets, either through the export tools (*i.e*. protein-DNA interactions) or through the TF page (*i.e*. TF-TF dimerization). Moreover, EDGEdb enables the export of interaction information for multiple genes or TFs at a time. Second, specific sequences bound by a TF or a set of TFs can be retrieved. This is very important for users that aim to analyze sequences that interact with a particular TF (*e.g *to computationally infer consensus TF binding sites). Third, sequence and gene identifiers in EDGEdb will be updated with every WormBase freeze. This is important as gene identifiers and coordinates may change, and because the conversion of old gene IDs to new gene IDs and coordinates may be tedious. Thus, the data within EGDEdb will always be compatible with the latest WormBase freeze. Finally, EDGEdb includes TF consensus binding sites and TF dimerization data from the literature where available. We will continue to include protein-DNA interaction data from other sources in the future, as long as precise DNA sequence information is available for interacting genomic regulatory sequences. We encourage researches to send us their interaction data that involve *C. elegans *TFs.

## Conclusion

EDGEdb provides a database for the storage, retrieval and integration of gene-centered protein-DNA interactions between *C. elegans *regulatory genome sequences and TFs; information regarding TFs; and differential gene expression data. This database will provide a valuable resource and tool for both the *C. elegans *and the systems biology community, and provides a framework to create similar databases for other organisms, including humans.

## Availability and requirements

Project name: EDGEdb a transcription factor-DNA Interaction database for the analysis of *C. elegans *differential gene expression

Project home page: 

Operating system(s): platform independent

Programming language: Java

Licence: the database is freely available to academic and non-academic users

## Abbreviations

Transcription factor (TF), open reading frame (ORF), untranslated region (UTR), green fluorescent protein (GFP), *C. elegans *differential gene expression database (EDGEdb).

## Authors' contributions

M.I.B, P.V. and F.C. created the database, under supervision of L.J. and A.J.M.W. The manuscript was written by M.I.B. and A.J.M.W.
